# Vascular leiomyoma in the oral cavity – Report of two cases

**DOI:** 10.4317/jced.55684

**Published:** 2019-06-01

**Authors:** Jaqueline-Lemes Ribeiro, Fernanda-Herrera Costa, Ana-Sueli-Rodrigues Cavalcante, Estela Kaminagakura, Yasmin-Rodarte Carvalho, Ana-Lia Anbinder

**Affiliations:** 1 Department of Bioscience and Oral Diagnosis, Institute of Science and Technology, São Paulo State University (Unesp), São José dos Campos, São Paulo, Brazil

## Abstract

Vascular leiomyomas (VL) are benign lesions of perivascular origin. We report two new cases and discuss their clinical, histological and immunohistochemical characteristics, in order to facilitate the diagnosis and treatment of such lesions. The patients, both male, presented asymptomatic nodules located in the bottom of the labial sulcus and buccal mucosa. In the second case, color doppler ultrasonography was performed, which showed no change in blood flow. After excisional biopsies, a limited lesion was observed histologically, with multiple tortuous vessels of varying sizes and calibers, and among them, spindle cells bundles, positive for smooth muscle actin. Oral VLs have clinical features similar to those of other more common lesions, making preoperative diagnosis difficult. Imaging examination, such as color doppler ultrasonography, may help in diagnosis. In general, excisional biopsy is performed, due to the ease of removal of the lesion during surgery. The treatment of choice is the complete excision of the lesion, which has an excellent prognosis and a low rate of recurrence.

** Key words:**Vascular leiomyoma, spindle cells, thrombus, Doppler.

## Introduction

Leiomyomas are benign neoplasms originating from smooth muscle, which occur, more commonly, in the female genital tract and rarely in the oral cavity (1). Clinically, it usually presents as a slow growing nodule of variable size. Histologically, they can be classified as solid, vascular or epithelioid. Vascular leiomyomas (VL) represent approximately 5% of all benign soft tissue tumors and present as a well-defined lesion, with vascular spaces associated to smooth muscle spindle cells often in concentric pattern (2). It is suggested that the origin of oral leiomyomas is the tunica media of blood vessels (3).

The aim of this study is to report two uncommon cases of oral vascular leiomyoma and to discuss their clinical, histological and immunohistochemical characteristics, in order to facilitate the diagnosis and treatment of such lesions.

## Case Report

**Figure 1:**
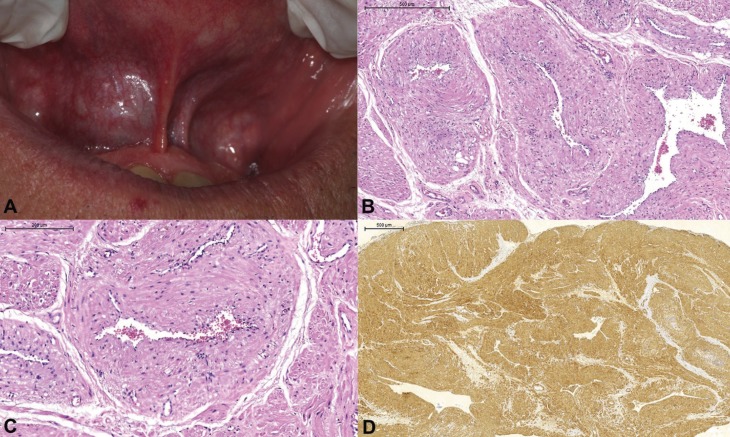
Case 1. (A) Submucosal nodule, similar in color to the adjacent mucosa, in the anterior region of the maxilla, right side of the patient; (B, C) Lesion exhibiting multiple tortuous vessels with thickened muscle layer; H&E; (D) neoplastic cell positivity to smooth muscle actin.

Case 01 – A 60-year old male patient presented to the dentist complaining of “swelling of the gums” in the upper anterior region, with 4 months of evolution. The extraoral examination revealed an effacement of the nasolabial fold and lifting of right nasal alar region. On intraoral examination, an asymptomatic 1cm submucosal nodule was observed filling the anterior maxillary labial fold, lateral to the midline. The lesion presented a rubbery consistency on palpation and similar coloration to the adjacent mucosa (Fig. 1A). With a clinical hypothesis of neurofibroma, excisional biopsy was performed.

**Figure 2:**
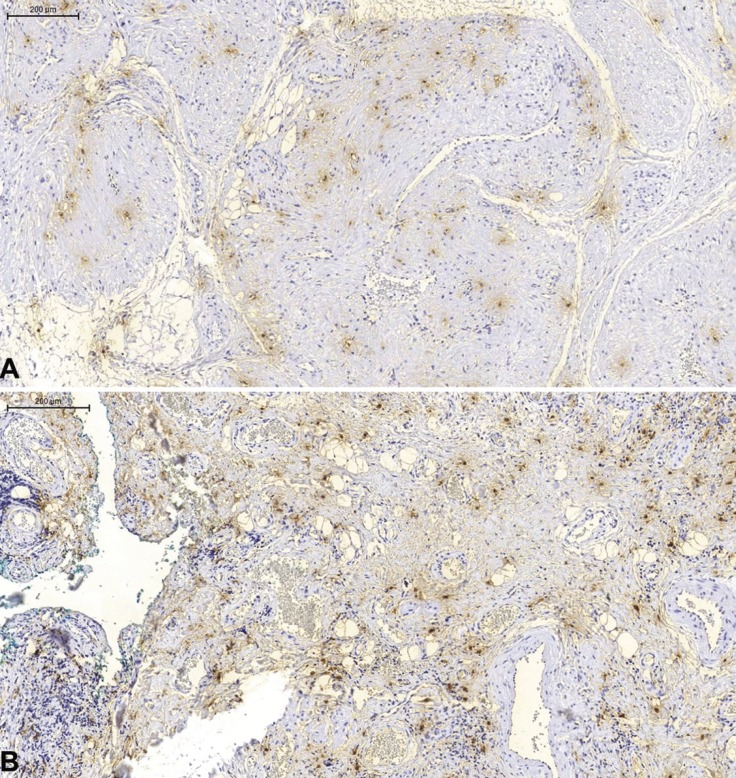
Immunohistochemical reaction (mast cell antibody) evidencing numerous mast cells in both cases. (A) case 1; (B) case 2.

Histological sections revealed a lesion containing multiple vessels with a thickened muscular layer (Fig. 1B,C). In general, the innermost muscular layers had a circumferential arrangement, while the outer ones showed an irregular pattern with bundles of spindle cells, sometimes interspersed with groups of adipose cells. Neoplastic cells exhibited pale-stained oval or blunt-ended nuclei and cytoplasmic vacuolization, sometimes perinuclear. The connective tissue stroma presented fibrous and myxoid areas. Neoplastic cells were positive to smooth muscle actin (Fig. 1D) (1A4; Dako; dilution 1:200) and negative to Bcl2 (124; Dako; dilution 1:50) and S100 antibodies (Dako; dilution 1:700). CD34 antibody (QBEnd 10; Dako; dilution 1:200) positively marked only the vascular endothelium. Numerous mast cells (AA1; Imgenex, dilution 1:700) could also be observed amid the lesion (Fig. 2A). The patient has been monitored for 2 years without recurrence.

**Figure 3:**
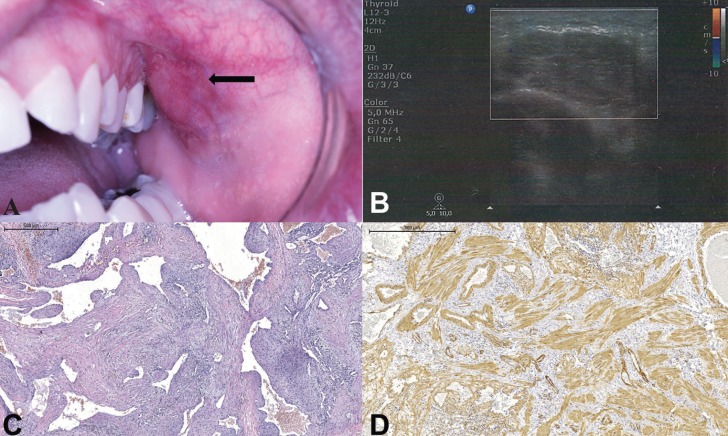
Case 2. (A) Submucosal nodule in buccal mucosa, left side (arrow); (B) Ultrasonography of the lesion, where an image of lobulated, hyperechoic area with well-defined limits can be seen, with no change in blood flow; (C) multiple thick-walled vascular spaces, surrounded by spindle cells; H&E; (D) neoplastic cell positivity to smooth muscle actin.

Case 02 – A 33-year old male patient attended our service complaining of “facial lesion”. Extraoral examination revealed a discrete facial asymmetry on the left side. On intraoral examination, the patient presented an asymptomatic submucosal nodule in the posterior buccal mucosa (Fig. 3A). The lesion was movable and presented a soft consistency on palpation. Under color Doppler ultrasonography, a well limited hyperechoic area was observed between cutaneous and muscular layers, with no change in blood flow (Fig. 3B). With the hypotheses of lipoma, solitary fibrous tumor and benign salivary gland neoplasia, excisional biopsy was performed. One week after surgery, the patient presented facial edema and intraoral bruising, which subsided after local physiotherapy.

Microscopically, a circumscribed lesion presenting multiple vascular spaces of various sizes and calibers, sometimes congested and interconnected was found. The cell lining of the vascular spaces consisted of a layer of endothelial cells, surrounded by spindle cells in layers of variable thickness (Fig. 3C). Sometimes these same cells were arranged in a more solid arrangement. An area of mixed thrombus in organization was observed, with formation of granulation tissue. The immunohistochemical reaction demonstrated positivity of neoplastic cells to smooth muscle actin (Fig. 3D) and negativity to CD34 antibodies, which marked only vascular endothelium. Mast cells could also be observed in abundance (Fig. 2B). The patient has been monitored for 6 months, without intercurrences or recurrence.

## Discussion

Leiomyomas are benign spindle cell lesions, commonly found in anatomical sites rich in smooth muscle, such as in the uterus and gastrointestinal tract, being very rare in the mouth due to poor smooth muscle presence in this region (2,3). In 2002, Brooks *et al.* (4) reviewed the literature, gathering 109 cases of oral leiomyomas already published in English. The most involved oral sites are lips, followed by palate, buccal mucosa and tongue. Silva *et al.* (5) reviewed the oral VL cases published between 2011 and 2016 and found 17 cases, with gingiva as the most common involved anatomical site. Of 14,056 cases diagnosed in our Oral Pathology service, within a 57-year interval, only 5 cases (0.036%) of VL were found ( Table 1), of which two have already been published (6). The mean age of our patients was 52.6 ± 12.52 years, slightly older than other series (4,5), with a predominance of males (3:2), in agreement with the data in the literature (4,5). Also, in the study by Brooks et al. (4), 76,412 biopsies of the oral pathology service were analyzed and only 12 cases were identified as VL (approximately 0.016%). In the study of Aitken-Saavedra *et al.* (7), VL corresponded to 0.08% of all benign oral tumors analyzed during the a 57-year period.

**Table 1:**
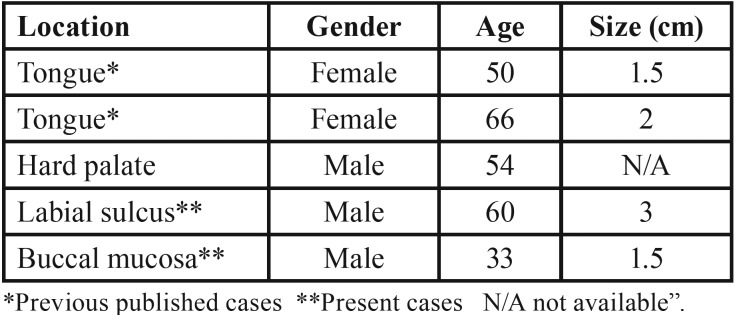
Cases of vascular leiomyoma in the Oral Pathology Archives of our Service, from 1962 to 2018.

The VL cases of our service, as well as most VL, were clinically asymptomatic, slow-growing submucous nodules (4), which had a mean size of 2±0.7cm. Due to their non-specific characteristics, other soft tissue lesions should be included in the clinical differential diagnosis, such as benign mesenchymal tumors, vascular and salivary gland lesions (4). Case 01 had as clinical hypothesis neurofibroma, and case 02, the diagnostic hypotheses were lipoma (due to the soft consistency), solitary fibrous tumor and benign salivary gland neoplasia, especially due to the depth of the lesion.

Vascular leiomyoma is a tumor that is difficult to diagnose clinically, but complementary tests may help. Ultrasonography (US) shows images with well-defined margins and homogeneous structure, which may indicate the benign nature of the lesion. The Doppler US usually suggests the presence of intratumoral vascularity (4). In case 02, however, a negative Doppler result was obtained, with no evidence of vascular alteration. This can be explained by the formation of thrombus and vascular obstruction which, after the biopsy, could be observed microscopically. In the literature, there is a description of few occurrences of thrombus formation, recent or organized, not being a common finding (2).

Microscopic diagnosis may also be difficult due to features common to other spindle cell lesions, such as neurofibroma, schwannoma or fibroma. In these cases, staining such as Masson’s trichrome and immunohistochemical reactions should be considered to investigate the origin of the lesions, and the positivity for smooth muscle actin confirms the smooth muscle origin of neoplastic cells. The malignant counterpart of leiomyoma, leiomyosarcoma, should be evaluated and ruled out. The morphological difference between them is the degree of cellular pleomorphism, nuclear atypia, hyperchromatism, necrosis and number of mitoses, which are the main aspects to be taken into account (4).

The abundant presence of mast cells in some areas of leiomyoma from the extra-peritoneal cavity in the lower abdomen, which appear to be related to angiogenesis and tumor growth, was reported previously (9). Although observed in the two cases presented, the presence or absence of mast cells has not been described in other oral VL.

Small traumas, venous stasis or hormonal causes have been suggested as the etiology of this lesion, however, it remains unknown (3,10). In some previously reported cases, the expression of progesterone and estrogen receptors has been evaluated. Some studies, performed in VL of the genital tract, nasal cavity, lung, oral cavity, and extraperitoneal cavity, demonstrated positivity only for progesterone receptors, and the others showed negativity for the two receptors (1). Of the two cases in the mouth where the expression of hormone receptors was evaluated, one was positive only for progesterone receptor, and another negative for both receptors (1,11). Therefore, the hormonal dependence of VL in the oral cavity is still quite controversial (1).

The treatment of choice for VL is complete surgical excision, with an excellent prognosis and few recurrences have been reported (3). Despite vascular involvement, excessive bleeding during surgery is not commonly observed (12).

In conclusion, oral VL have clinical features similar to those of other common lesions, making preoperative diagnosis difficult. Imaging examination, such as color Doppler ultrasonography, may help in diagnosis. In general, excisional biopsy is performed, due to the ease of removal of the lesion during surgery, and although it has vascular components, excessive bleeding during the procedure is not common. The treatment of choice is the complete excision of the lesion, which has an excellent prognosis and a low rate of relapse.
